# Prevalence and factors associated with burnout among health professionals of a public hospital network during the COVID-19 pandemic

**DOI:** 10.1371/journal.pone.0298187

**Published:** 2024-04-22

**Authors:** Patricia Martins, Richardson Warley Siqueira Luzia, Jair Alves Pereira Filho, Kelly Silva Welsh, Cíntia Fuzikawa, Rodrigo Nicolato, Márcia Mascarenhas Alemão, Márcio Augusto Gonçalves, José Carlos Cavalheiro, Ianny Dumont Ávila, Ricardo Teixeira Veiga

**Affiliations:** 1 Department of Mental Health, Faculty of Medicine, Federal University of Minas Gerais (UFMG), Belo Horizonte, Minas Gerais, Brazil; 2 Statistics and Workforce Management Center at Hospital Foundation of the State of Minas Gerais (FHEMIG), Belo Horizonte, Minas Gerais, Brazil; 3 Ezequiel Dias Foundation (FUNED), Belo Horizonte, Minas Gerais, Brazil; 4 Department of Health Management, School of Nursing, Federal University of Minas Gerais (UFMG), Belo Horizonte, Minas Gerais, Brazil; 5 Department of Administrative Sciences, Faculty of Economic Sciences, Federal University of Minas Gerais (UFMG), Belo Horizonte, Minas Gerais, Brazil; 6 Department of Letters, Pontifical Catholic University of Minas Gerais, Belo Horizonte, Minas Gerais, Brazil; 7 Academic at the Faculty of Medical Sciences of Minas Gerais, Belo Horizonte, Minas Gerais, Brazil; UFPE: Universidade Federal de Pernambuco, BRAZIL

## Abstract

Burnout is most commonly defined as a syndrome characterized by emotional exhaustion, cynicism, and ineffectiveness, which occurs in response to chronic stressors at work. It can adversely affect health workers’ physical and mental health, and the quality of care provided. The COVID-19 pandemic increased stressors and could impact burnout prevalence in this group. There is a lack of information regarding the prevalence of burnout among hospital health workers in Brazil. A newer definition of burnout has been proposed that considers three different clinical profiles: the frenetic, underchallenged and worn-out subtypes. This differentiation could lead to interventions tailored for each subtype. The present study aimed to estimate the prevalence of burnout, its subtypes, and associated factors in workers of a public hospital network in Brazil, during the pandemic. A total of 143 randomly selected participants answered an online form that included sociodemographic and occupational items, and the Burnout Clinical Subtypes Questionnaire, a summarized version. This questionnaire evaluates three burnout dimensions (overload, lack of development, neglect) that can be used to discriminate the three burnout subtypes (frenetic, underchallenged, worn-out, respectively); higher scores indicate higher burnout levels. The prevalence of burnout was high (53.85%), similar to other studies during the pandemic. The most common subtypes were ‘frenetic’ (34.97%), characterized by increased efforts to meet work demands, to the point of neglecting personal needs, and ‘lack of development’ (23.78%), characterized by a sense that work is uninteresting and does not contribute to personal development, and a perfunctory behavior towards tasks. Age was associated with burnout: workers with less than 51 years presented higher levels of burnout. These findings indicate the need for effective interventions to prevent and/or treat burnout. The assessment of burnout subtypes can allow managers to better understand the processes affecting employees, and inform actions to improve workforce health.

## Introduction

Burnout has been conceptualized as a syndrome that occurs as a response to prolonged stressors in the work environment. The Maslach Burnout Inventory (MBI), which operationalizes the most commonly used burnout definition, identifies emotional exhaustion, cynicism, and ineffectiveness as the syndrome’s three main dimensions. Exhaustion refers to the feeling of being depleted of emotional resources, having no longer any to offer; cynicism is described as detached, harmful, or even callous behavior towards work; and inefficiency, as feelings of inadequacy and incompetence resulting in reduced self-efficacy and personal fulfillment [1, 2].

Among health professionals who work in hospitals, the prevalence of burnout can reach 25–33% in intensive care nurses and 45% in intensive care physicians [3, 4], with up to 86% presenting at least one of the three dimensions [5]. Besides work overload, time pressure, and administrative burden, factors contributing to burnout among hospital workers include frequent exposure to suffering, death, and social inequalities, and less autonomy due to complex management [6–8]. In addition to the impact on physical and mental health, burnout can result in absenteeism, decreased productivity, worsening of clinical activities, lower patient satisfaction, reduced quality of care, and medical errors [4, 9].

The COVID-19 pandemic increased stressors for health professionals, such as work overload, exposure to the risk of infection and death, fear of infecting others, and the need to self-isolate, besides the disruption of daily activities. Systematic reviews and meta-analyses show that during the pandemic, nearly half of the health workers suffered from burnout [10], and among physicians, the pooled prevalence was 43.6% [11]

There have been several studies on the prevalence of burnout among hospital health workers in Brazil before the COVID-19 pandemic. However, most evaluated specific sectors of the hospital or professional categories. Intensive care units and nurses have been the most studied [12, 13]. Therefore, there still needs to be more information regarding burnout among hospital workers in general.

In Brazil, the mediocre economic growth in previous years and severe cuts in social programs undermined the effectiveness of the public Brazilian healthcare system in facing the COVID-19 pandemic [14]. Furthermore, the Brazilian federal government’s denialism delayed the start of mass vaccination, raised the number of severe cases and deaths, and hindered the collection of reliable health data through the network of public institutions. In fact, among the 20 countries most affected by the pandemic, Brazil has the fourth highest mortality rate per 100,000 inhabitants [15], and there have been more than 700,000 deaths due to COVID-19 in the country [16]

Prospective studies suggest that the pandemic may have caused burnout rates to rise. For example, one study found that the rate of burnout among emergency and intensive care unit (ICU) workers rose from 19.5% to 32% during the pandemic [17], and another study found that the rate of burnout among ICU doctors doubled from 2017 to 2018 (37% to 57%) [18]. The increased burnout among health professionals during the pandemic from levels that were already high, its impact on mental and physical health, and the undesirable effects on patient care underscore the importance of increasing efforts to understand, treat, and prevent this syndrome. Farber’s idea of three subtypes of burnout, each with ways of treating and preventing it, could help us understand this context better and decide what to do [19].

Farber argued that burnout was not a uniform entity with similar symptoms across individuals, as the traditional approach suggests. Based on his clinical experience, he proposed three subtypes of burnout: frenetic, underchallenged, and worn-out. In the frenetic subtype, individuals are highly involved in work to the point of neglecting their needs. They are ambitious and have difficulty acknowledging failures. When faced with obstacles at work, they tend to increase their efforts to reach the desired outcomes. Individuals in the underchallenged subtype have lost interest in work and carry on tasks superficially but pay attention to them. They think their current work hinders their personal development by not promoting the use of their talents and capacities. Difficulties at work are met by reducing energy and enthusiasm, thus increasing detachment. In the worn-out subtype, individuals have reduced their involvement with work to the point of neglecting their responsibilities. When encountering difficulties at work, they give up [19].

The Burnout Clinical Subtype Questionnaire (BCSQ-36) was the first tool used to define these subtypes of burnout syndrome [20]. Later, a shorter version with only 12 items was available (BCSQ-12) [21]. Both versions have shown good internal consistency, validity, and reliability and have been validated in Brazil [20–22].

To the best of our knowledge, this is the first study to evaluate the prevalence of burnout syndrome, its subtypes, and associated factors among workers of all categories in a hospital network using the BCSQ-12.

## Objective

The present study aimed to estimate the prevalence of burnout syndrome and its three subtypes among employees of a Brazilian public hospital network during the COVID-19 pandemic. The study also evaluated the association of burnout with sociodemographic and occupational factors.

## Methods

An exploratory, descriptive, cross-sectional study with a quantitative approach was performed using a self-reported online questionnaire.

### Participants

The Hospital Foundation of the State of Minas Gerais (FHEMIG) is a network of public hospitals run by the government of the State of Minas Gerais, Southeast Brazil. The study population consisted of all employees of nineteen hospitals of the FHEMIG network, eighteen of which are located in the state capital, Belo Horizonte. Exclusion criteria were the presence of acute depression (less than six months), schizophrenia or psychotic disorders, and the use of medications that cause cognitive impairment, considering that these conditions could interfere with the quality of the information given. Additionally, depression could be a confounder of burnout due to the similarity between symptoms of both conditions.

The sample size was calculated considering the total population of 10,983 workers, a 95% confidence level, a 3.5% margin of error, and an estimated prevalence of burnout syndrome of 10%, leading to a sample of 283 individuals. Participants were selected using simple random sampling.

### Data collection / instruments

Data collection occurred between April and October of 2021. Individuals selected through the simple random sampling received an email that explained the study, invited them to participate, and contained a link to an online form. The email also stated the exclusion criteria and requested individuals who fulfilled any of those criteria contact the research team. When there was no response, the same email was sent again. If there still was no response, another participant was randomly selected.

The first part of the online form was a term of free, informed consent, and only responses from participants who provided their informed consent were considered. The rest of the form was a self-reported questionnaire that included:

Sociodemographic and occupational items: age, sex, if the participant had a stable partner, number of children, professional category, occupation, years of work at FHEMIG, years of work in current position, type of employment contract, nature of employment (full or part-time), financial difficulties, medical leave during the last year, and direct involvement in the care of patients with COVID-19;

The Burnout Clinical Subtype Questionnaire, summarized version (BCSQ-12) [23]. This questionnaire was developed from the original 36-item questionnaire (BCSQ-36). The BCSQ-36 assesses the presence of the three subtypes of burnout syndrome that Farber identified: frenetic, underchallenged, and worn-out. Three dimensions of each subtype are measured. For the frenetic subtype, the dimensions are overload, meaning the feeling of being overwhelmed, caused by neglecting health and personal life in favor of work; grandiosity, as the need for achievements and difficulty to acknowledge failures; and a high degree of involvement in work, with increasing efforts when faced with difficulties. For the underchallenged subtype, the dimensions assessed are lack of development, related to the feeling that one’s talents are not being recognized and used in work, which can even lead the individual to consider changing jobs; indifference, meaning working in a superficial and disinterested way, although not neglecting most responsibilities; and boredom, as experiencing work as monotonous. The dimensions of the worn-out subtype that the BSCQ-36 assesses are neglect, the lack of involvement in tasks that leads to giving up when faced with difficulties; lack of acknowledgment, referring to the feeling that the organization one works for does not acknowledge effort or dedication; and lack of control, related to the feeling that the difficulties faced at work prevent tasks from being done satisfactorily and are not under one’s control [20].

The BSCQ-12 evaluates three dimensions: overload, lack of development, and neglect. These dimensions were better at telling the difference between the three types of burnout. They were also more similar to the dimensions of the MBI, which is the most common tool used to measure burnout. The dimensions of overload, lack of development, and neglect relate to the classic MBI dimensions of emotional exhaustion, cynicism, and ineffectiveness, respectively. In this sense, the BSCQ-12 could provide information regarding the typological and standard approaches to burnout syndrome [21]. The BSCQ-12 has shown good construct and criterion validity and has been validated in Brazil [22].

Four statements evaluate each dimension in the BSCQ-12. Participants should indicate their agreement with each statement using a 7-point Likert-type scale from 1 (strongly disagree) to 7 (strongly agree) [21].

### Statistical analysis

The software used for data analysis was the Statistical Package for the Social Sciences (SPSS)® version 20 and R [24].

Sociodemographic and occupational characteristics were described using means and standard deviations for age and percentages for other variables. Subsamples were compared using test χ2. For quantitative variables, the normality test was carried out.

Confirmatory factor analysis (CFA) was used to examine the factor structure of the BCSQ-12 [25].

The score in each dimension of the BCSQ-12 was the average number of points attributed to the four statements that evaluated that dimension. Higher scores meant higher levels of burnout. Burnout syndrome was considered present when the score in at least one of the three dimensions was above the 75th percentile for that dimension. Thus, for the overload dimension, the cut-off score was >5; for lack of development, >4.5; and for neglect, >3.

Logistic regression (LR) examines the association of one or more independent variables with one dichotomous dependent variable. LR was used to evaluate the association of burnout syndrome (dependent variable) with age, sex, professional category, years of work at FHEMIG (dichotomized as less than or more than 9 years), type of employment contract, medical leave during the last year, and direct involvement in the care of patients with COVID-19 (independent variables).

Multiple factor analysis (MFA) is a multivariate technique to summarize and visualize a complex data table [26] based on principal components and multiple correspondence analyses. MFA was applied to compare the relative importance of the three burnout syndrome dimensions and corresponding variables to explain the variance in the burnout score.

### Ethical considerations

The research was conducted following the Declaration of Helsinki of 1975, revised in 2013, and was authorized by the FHEMIG Research Ethics Committee (Technical Opinion No. 4.480.128/2020). All participants provided free informed consent.

## Results

Due to the low response rate (4.45%), emails were sent to 3,214 employees. From the expected sample of 283 participants, a final sample of 143 participants was obtained. This sample size allowed for a confidence interval of 90% and a margin of error of 5%.

The participants’ sociodemographic and occupational characteristics are presented in Table 1. The average age was 41.78 ± 9.01 years, and most participants were women (78.3%), nurses (31%), who were working for more than six years at FHEMIG (81.9%), and statutory permanent employees (81%).

**Table 1:** Participants’ sociodemographic and occupational characteristics (n = 143).

Characteristic	n	%
**Age (years)**		
< 35	29	20.4
35–50	86	60.6
> 50	27	19.0
**Women**	112	78.3
**Has a stable partner**	98	68.5
**No children**	58	40.6
**Professional category**		
Nursing	44	30.8
Medicine	21	14.7
Other	78	55.5
**Years working at the institution**		
< 6	26	18.2
6–10	65	45.5
> 10	52	36.4
**Years working in the current position**		
< 5.1	67	47.2
5.1–10	47	33.1
> 10	28	19.7
**Type of contract**		
Temporary	24	16.8
Permanent	3	2.1
Statutory permanent employee	116	81.1
**Nature of employment**		
Full-time	106	74.1
Part-time	37	25.9
**Financial difficulties**		
Never	24	16.8
Sometimes	71	49.7
Many times	23	16.1
Most of the time	9	6.3
Always	16	11.2
**Medical leave in the last 12 months**		
Yes	56	39.2
No	87	60.8

The presumed BCSQ-12’s 3-factor structure suited well the sample data, according to CFA (normed chi-square = 1.84, CFI and TLI > 0.97, and RMSEA < 0.08) [25]. Moreover, its dimensions were reliable and valid.

The prevalence of burnout was 53.85% (n = 77). Table 2 shows the prevalence of burnout according to the three dimensions evaluated by the BCSQ-12. The total prevalence of the dimensions of overload, lack of development, and neglect were 34.97%, 23.78% and 12.59%, respectively.

**Table 2:** Prevalence of burnout according to dimensions evaluated by the BCSQ-12 (n = 143).

Dimension(s)	n	%
Overload only	33	23.07
Lack of development only	17	11.89
Neglect only	5	3.50
Overload and lack of development	9	6.29
Overload and neglect	5	3.50
Lack of development and neglect	5	3.50
Overload and lack of development and neglect	3	2.10
Total (any dimension present)	77	53.85

Table 3 presents the prevalence of burnout according to sociodemographic and occupational characteristics. Age was the only variable associated with burnout (*p*-value = 0.007).

**Table 3:** Prevalence of burnout syndrome according to sociodemographic and occupational characteristics^a^.

Characteristic	Burnout		Total	Prevalence (%)	*p*-value
	No	Yes			
**Age (years)**					0.01*
<30	11	18	29	**62.0**	
31 to 50	35	51	86	**59.2**	
>51	19	8	27	**29.6**	
**Sex**					0.77
Male	16	16	31	51.6	
Female	51	61	112	54.4	
**Professional category**					0.07
Physician	13	9	22	40.9	
Nursing	27	23	50	46.0	
Other	26	45	71	63.8	
**Years of work at the institution**					0.9
>9	58	67	125	53.6	
<9	8	10	18	56.4	
**Type of Contract**					0.81
Temporary or non-statutory	13	14	27	51.8	
Permanent Statutory	53	63	116	54.3	
**Direct care of COVID-19 patients**					0.67
Yes	28	38	58	51.7	
No	38	47	85	55.3	
**Medical leave in the last 12 months**					0.52
Yes	24	32	56	57.1	
No	42	45	87	51.7	

^a^ Mann-Whitney test

Logistic regression showed that age was the only variable associated with burnout (Table 4).

**Table 4:** Logistic regression analysis^a^ of characteristics associated to burnout.

Characteristic	β	Exp(β)	95% CI for Exp(β)		*p*-value
			Lower	Upper	
**Age (years)**					
>51	1				
<30	1.528	4.608	1.144	18.557	**.032**
31 to 50	1.145	3.143	1.169	8.453	**.023**
**Sex**					
Female	1				
Male	-.046	.955	.398	2.289	.918
**Professional category**					
Other	1				
Physician	-1.011	.364	.111	1.190	.095
Nursing	-.844	.430	.173	1.070	.070
**Years of work at FHEMIG**					
>9	1				
<9	-.105	.901	.209	3.872	.888
**Type of Contract**					
Statutory	1				
Temporary or non-statutory	-.217	.805	.262	2.474	.704
**Direct care of COVID-19 patients**					
No	1				
Yes	.146	1.157	.493	2.719	.738
**Medical leave in the last 12 months**					
No	1				
Yes	.244	1.276	.605	2.694	.522

^a^ Wald test

In the MFA of the burnout construct, the eigenvalues showed that the two first principal components accounted for more than 65.76% of its variance. ‘Lack of development’ explained 40.4% of the first principal component, but its importance was negligible in the second one (<1%), in which the dimensions ‘overload’ (54.7%) and ‘neglect’ (44.9%) were the most relevant.

## Discussion

The burnout prevalence in this sample of employees of a public hospital network in Brazil during the COVID-19 pandemic was 53.84%, Age was the only variable associated with burnout among the sociodemographic and occupational characteristics studied. The BCSQ-12 dimensions ‘overload’ and ‘lack of development’ were the most prevalent.

The level of burnout witnessed is comparable to that found in a study of healthcare workers (HCWs) during the first year of the pandemic, where 52% of them experienced burnout [10] It is also similar to a study conducted on professionals in Brazil who were caring for COVID-19 patients in hospitals, where 48% of them experienced burnout [27]. These percentages undoubtedly indicate high levels of burnout.

Hospital environments may present some characteristics, such as limited flexibility, autonomy, and voice; a lack of a culture of collaboration and vulnerability; limited time with patients and colleagues; an absence of focus on health worker well-being; and contact with suffering and death, that contribute to burnout [28].

During the pandemic, work overload, fear of contamination, and disruption of daily life may have aggravated this unfavorable situation. As the pandemic became politicized, some healthcare workers faced hostility, threats, and violence often related to misinformation about the virus. Therefore, since the beginning of the pandemic, it was assumed that burnout among healthcare workers would increase even more than in the general population [29]. Indeed, a systematic review of four studies by Sanghera et al. concluded that direct exposure to Covid-19 patients was the most common risk factor identified for all mental health outcomes, including occupational burnout [30]. Notably, these data are close to those already found in studies before the pandemic [31, 32], which indicates the diversity of factors involved in this syndrome.

The impact of the COVID-19 pandemic on the levels of burnout found in the study sample is unclear. Although some longitudinal studies have indicated an increase compared to pre-pandemic levels [33] and an increase in stressors possibly happened in the study population, there was no previous data on burnout levels in workers of this public hospital network for comparison purposes. The fact that burnout prevalence was not associated with being in direct care of COVID-19 patients does not preclude the possibility of the impact of the pandemic because both frontline and non-frontline COVID-19 workers experienced burnout during the pandemic [10].

Among the sociodemographic and occupational factors studied, younger age (<50 years) was associated with a higher prevalence of burnout. A recent scoping review on factors related to burnout among HCW during the pandemic found mixed results. Many studies did not find an association between age and burnout, and when there was an association, most frequently burnout was associated with younger age, as in the present study [34].

Factors that could contribute to higher levels of burnout in younger workers include fewer skills to handle stressful situations related to frequent frustrations and work demands, which generally trigger insecurity about knowledge and position, contributing to the emergence of the syndrome. This situation may have increased in number and intensity during the pandemic. Younger workers may also need to juggle work–family conflicts, another stressor that could contribute to burnout. Other possibilities would be that workers in the older group have less stressful tasks than the younger group or that there has been a selection over time, and their age peers who had burnout changed jobs or had to leave due to health problems [34, 35]. Nevertheless, the relationship of work characteristics and age with occupational well-being remains complex [36].

Regarding the BCSQ-12, it can be used to discriminate the subtype of burnout, according to Farber’s typology: ‘overload’ corresponds to the frenetic subtype, ‘lack of development’, to the underchallenged, and ‘neglect’, to the worn-out subtype [21]. Therefore, in the sample studied, the frenetic subtype was the most prevalent, followed by the underchallenged and worn-out.

Individuals presenting the frenetic subtype of burnout are highly involved with their jobs and work harder when faced with challenges. This behavior can lead to overload, imbalance between work and private life, and jeopardize their health. The choice to be an HCW frequently stems from a desire to help, to give of oneself to relieve the suffering of others. It is not surprising that individuals with this kind of motivation tend to be highly involved with work and to increase efforts to meet demands to the extent they overlook their own needs. The pandemic could have contributed to intensifying this commitment to work.

On the other hand, it could also have increased the stress and overload of HCW. Such a condition could explain why this subtype was the most prevalent in the sample studied. As the frenetic type is often the first manifestation of burnout, early detection and intervention could prevent the syndrome’s progression [37].

In the underchallenged subtype individuals experience work as monotonous and unstimulating. They perform their tasks perfunctorily and may have the feeling of being trapped in their work. It is more frequent seen in jobs with repetitive tasks, but there is also a component of how the person feels about the job. Most workers in the study were permanent statutory employees. They are admitted after a very competitive public selection process, and after a probation period, they become permanent employees. This situation could contribute to staying the same job even if one finds it unsatisfying. This subtype could also develop after the frenetic subtype, when frustration due to not meeting expectations, even with additional effort, leads to a reduction in dedication to work [37].

The worn-out subtype was much less prevalent. It is characterized by omission, neglect and disengagement in response to chronic stressors at work. Affected individuals may have feelings of hopelessness and incompetence. From a longitudinal point of view, it may develop after the underchallenged subtype, representing a further reduction in dedication to work. The low prevalence found may reflect the commitment of HCW to their activities, which may even have increased during the pandemic. The answers to the questions that refer to this subtype may also have been affected by social desirability, leading to an underestimation of the actual prevalence [37].

The high level of burnout observed in the present study and others and the harmful consequences of this syndrome highlight the need for prevention and interventions for burnout in HCW. In this regard, preventive organizational strategies are generally more effective than ameliorative interventions focusing on the individual. Recommended organizational strategies include appropriate adjustments and regular review of work schedules, with sufficient rest time between shifts, adequate facilities, and services, education about burnout and its consequences, and peer support [38]. A recent study of primary care workers during the pandemic illustrates the impact of organizational factors. Despite high levels of burnout, with 68% presenting features of the frenetic subtype, having received specific training about COVID-19 and feeling involved in decision-making were associated with lower levels of burnout [39].

Individual interventions can help workers to deal with stress and reduce burnout. Most use one or a combination of relaxation, cognitive behavioral or mindfulness-based approaches, and education on burnout and stress management [37, 40]. In Brazil, the integrative and complementary practices constituting the National Policy on Integrative and Complementary Practices within the Brazilian Unified Health System (SUS) scope have been the most studied [41, 42].

There are no official guidelines as to which individual intervention would be more effective for which professional category or individual [37]. However, one of the reasons for proposing the three subtypes of burnout was the realization that burnout was not a uniform entity but could present different clinical profiles, which called for specific individual intervention strategies. Therefore, in the case of the population of this study, interventions addressing the frenetic and underchallenged subtypes may be considered appropriate, considering the high prevalence of these subtypes.

In the frenetic subtype, individuals present a state of chronic overactivation, with tension that can lead to exhaustion. So, interventions to reduce the levels of physiological arousal, such as relaxation and meditation, could be helpful. Mindfulness-based programs have been proposed to increase the awareness of how the person deals with increased demands and what underlies this pattern of behavior, and also help develop alternative strategies to deal with this kind of situation. In the underchallenged subtype, clarifying the person’s values and goals might help explore possibilities of new challenges that would carry personal meaning. These changes may renew interest and engagement in work [37].

The small number of participants is a substantial limitation of this research. Although the expected response rate in webmail surveys is historically low, the typical work overload in Brazilian public hospitals and the high levels of burnout may have made employees less available or receptive to participate in the study. The small sample may have resulted in the underestimation of burnout prevalence. Another limitation is that the cross-sectional study design allows only the identification of factors associated with burnout.

Longitudinal studies could increase the knowledge of risk factors for burnout. Moreover, there is a need for follow-up studies that evaluate the effect of interventions to prevent and/or treat burnout. The assessment of the prevalence of burnout subtypes can allow managers to better understand the processes that are affecting employees, and inform actions, on the organizational and individual levels, to improve workforce health and promote a healthy work environment. Even if COVID-19 is currently no longer a public health emergency, these actions are called for, not only because the possibility of future health emergencies cannot be eliminated, but because the pandemic was one of many factors that can contribute to the high prevalence of burnout.

## Conclusions

The estimated prevalence of burnout in this sample of workers of a network of public hospitals in Brazil during the COVID-19 pandemic was high (53.85%), and the group with less than 51 year of age presented higher levels. Considering the burnout subtypes assessed by the BCSQ-12, ‘frenetic’ (34.97%) and ‘lack of development’ (23.78%) subtypes were the most prevalent.
